# Lateral transperitoneal laparoscopic adrenalectomy: A single-centre experience of 21 procedures

**DOI:** 10.1515/med-2025-1230

**Published:** 2025-09-06

**Authors:** Saman Anwar Wahid, Shakhawan Hama Amin Said, Lusan Abdulhameed Arkawazi, Mzhda Sahib Jaafar, Zhino Nuri Hussein, Attar Abdul Kareem Nadir

**Affiliations:** Department of Urology, Sulaimani Teaching Hospital, Sulaimani Directorate of Health, 0046, Sulaimaniyah, Iraq; Branch of Clinical Sciences, College of Medicine, University of Sulaimani, Sulaimaniyah, Iraq; Department of Surgery, Shar Teaching Hospital, Sulaimani Directorate of Health, Sulaimaniyah, Iraq

**Keywords:** laparoscopic technique, lateral transperitoneal adrenalectomy, functioning adrenal tumours, nonfunctioning adrenal mass

## Abstract

**Background:**

Laparoscopic adrenalectomy (LA) is the gold standard technique for well-selected patients with an adrenal mass. The surgical team’s experience is an essential factor in the surgical outcome.

**Objective:**

The aim of this study was to assess the outcomes of patients who underwent lateral transperitoneal LA to remove their adrenal mass.

**Methods:**

In this case-series study, 20 consecutive patients with adrenal mass were enrolled in Sulaimani Teaching Hospital, Sulaimaniyah, Iraq, from March 2019 to March 2022. Patients underwent lateral transperitoneal LA under general anaesthesia. Then, the type of LA, operation time, mass size, hospital stay, histopathological finding, and postoperative complications were assessed.

**Results:**

Most patients were females (65%), underwent left-sided LA (55%), had a nonfunctioning mass (55%), and had no postoperative complications (85%). The mean operation time was 95 ± 2.0 min, and the mean hospital stay was 3.2 ± 1.5 days. Most patients were detected incidentally through radiology (55%), while others were detected after biochemical tests were done, including cases of pheochromocytoma (25%), hypercortisolism (10%), and hyperaldosteronism (10%). Most patients had benign cortical adenoma (45%), followed by benign medullary pheochromocytoma (25%), myelolipoma (15%), oncocytoma, adrenal hyperplasia, and ganglioneuroma (5.0% each).

**Conclusions:**

LA seems to be a safe and feasible option for selected cases in this locality.

## 1 Introduction

Adrenal tumours are very commonly encountered in the practice of radiology. They may arise from the adrenal gland (the cortex or the medulla) or be secondary lesions. They may be benign or malignant. Clinically unapparent adrenal tumours (≥1 cm in diameter) are found in 2–10% of the population worldwide, known as adrenal incidentaloma, which is commonly discovered incidentally on cross-sectional abdominal imaging performed for reasons other than adrenal mass [1]. The incidence of adrenal tumours increased 10-fold in the past two decades, with most diagnosed in older adults. Malignancy is diagnosed in 5–8% of patients with adrenal tumours, with a higher risk in young patients [2].

Functioning adrenal tumours lead to hypersecretion of adrenal hormones (mainly cortisol and aldosterone) and result in clinical syndromes. In contrast, nonfunctioning (inactive) adrenal adenomas do not produce excess adrenal hormones and comprise most adrenal adenomas. The latter do not cause symptoms or require treatment [3,4]. Computed tomography (CT) and positron emission tomography are the most common imaging modalities for the initial evaluation of adrenal tumours. Magnetic resonance imaging (MRI) and functional scintigraphic techniques are frequently used for atypical presentations or further assessment. Additionally, molecular tests such as F-18 FDG, I-123 metaiodobenzylguanidine, and In-111 Octreotide imaging are essential in the diagnostic algorithm of the adrenal masses [5].

Laparoscopic adrenalectomy (LA) was first described through a transperitoneal approach by Gangar in 1992 as a surgical treatment for most adrenal tumours. Since then, LA has been regarded as a gold standard technique for small benign lesions and adrenal masses [6]. Malignant adrenocortical tumours are the leading cause of open surgery to avoid the dissemination of cancer. Tumour size is essential for decision-making, but no consensus exists for open surgery indication. However, open adrenalectomy (either via a large flank or abdominal incision) is associated with high morbidity, especially in obese patients. At the same time, LA appears to achieve superior results regarding recovery, cosmetics, and morbidity [7].

Moreover, LA has shorter operative time, less postoperative pain, less need for analgesia, early hospital discharge, and return to daily activity [7,8]. Most surgeons prefer the transperitoneal approach due to the ample and familiar working space and clear anatomic landmarks. Still, it carries more risk of visceral injury and postoperative pain than the retroperitoneal approach [9]. Thus, we used lateral transperitoneal LA to remove the adrenal mass and studied its postoperative outcomes among patients.

## 2 Materials and methods

### 2.1 Study design and setting

The case-series prospective study was performed on 20 consecutive patients with an adrenal mass in Sulaimani Teaching Hospital, Sulaimaniyah, Kurdistan region, Iraq, from March 2019 to March 2022.

### 2.2 Inclusion criteria

Patients with functioning and nonfunctioning adrenal mass (<100 mm diameter) and without features of malignancy were included, regardless of age and gender.

### 2.3 Exclusion criteria

Patients with other malignancies rather than adrenal mass and those with multiple endocrine neoplasia type 2 (MEN 2a) and subclinical Cushing syndrome were excluded.

### 2.4 Diagnosis

All adrenal masses were radiologically assessed by abdominal ultrasonography, CT, and MRI when required for further identification of the radiologic features of the tumours. Patients’ sociodemographic data were collected, including age, gender, and body mass index (BMI, kg/m^2^). Then, 5.0 mL of venous blood was collected from each patient for biochemical tests. Plasma-free cortisol was done to exclude subclinical Cushing syndrome. Patients with features of hypercortisolism were further assessed by measuring 24 h urinary free cortisol, dexamethasone suppression test, and plasma adrenocorticotropic hormone level when indicated. Whereas patients presented with hypertension were investigated for plasma metanephrines, renin, aldosterone, and renin/aldosterone ratio subsequently. Patients with suspected pheochromocytoma were additionally examined for parathyroid hormone, and serum calcium was used to exclude MEN 2a.

### 2.5 Preoperative preparation

Preoperative medications were tailored to patient’s needs and tumour functionality. Patients with Cushing syndrome were given appropriate antihypertensive medication, and all were shifted to the appropriate insulin subtype injection the day before the operation. For pheochromocytoma cases, blood pressure was controlled over 2 weeks with increasing doses of Doxazosin tablets, followed by adding propranolol for another 2 weeks to control reflex tachycardia. In cases of Cohn’s syndrome, the blood pressure was controlled using an appropriate dose of spironolactone, and one of our cases presented with severe hypokalaemia (2.2 mEq/L) that required intravenous infusion of potassium over 48 h preoperatively, and another 80 mEq was given on the first postoperative day. All patients had routine haematologic, virology investigations, medical, and cardiologic assessment.

### 2.6 Surgical procedure

All patients received general anaesthesia, prophylactic antibiotics, and a central venous cannula and an arterial access cannula were established intraoperatively. All patients received one dose of hydrocortisone (100 mg, intravenously), which was repeated every 8 h if needed, and they were placed in the corresponding lateral decubitus position; the operation table was bent 40–60° at the level of the flank. We did not report any intra-operative hemodynamic instability; thus, no case has received any vasoactive medication intra-operatively. Consequently, 21 LA were done for 20 consecutive patients; then they were followed up for different durations based on the severity of the cases. Finally, the type of LA (left, right, and bilateral), operation time (minutes), mass size (mm), hospital stay (days), histopathological findings, and postoperative outcomes were assessed among operated patients. Then, they were followed up for at least 1 year, at 3-month intervals.

### 2.7 Statistical analysis

The statistical analysis was conducted using the IBM Statistical Package for the Social Sciences (SPSS, Chicago, USA, version 25). The data were presented in frequency and percentages with mean ± standard deviation (SD).


**Ethical approval:** All procedures, including patient selection and data collection, were approved by the Ethical Committee of the College of Medicine, University of Sulaimani, Sulaimaniyah, Iraq (No. 20/2 on January 20, 2019). The Declaration of Helsinki was followed throughout all study phases to ensure the highest level of ethical conduct.
**Informed consent:** Before the surgery, the patients provided written informed consent, and the surgical procedure and perioperative outcomes were discussed with them.

## 3 Results

**Figure 1:**
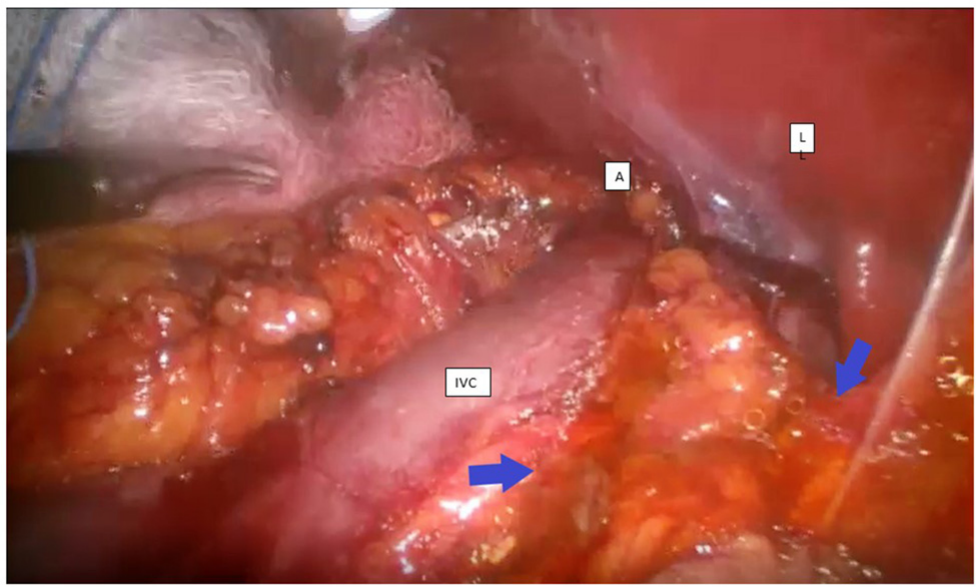
Intraoperative bile leak (arrows) is seen medial to the inferior vena cava, the adrenal gland (A), and below the caudate lobe of the liver (L).

The patients’ mean age was 39.9 ± 2.1 years, ranging from 23 to 56 years. Seven (35%) were males, and 13 (65%) were females. The mean BMI was 29.5 ± 1.1 kg/m^2^. Most patients underwent left-sided LA (*n* = 11, 55%), followed by proper LA (*n* = 8, 40%), and only one case (5.0%) underwent bilateral adrenalectomy. The mean operation time was 95 ± 2.0 min, ranging from 45 to 160 min, and the mean hospital stay was 3.2 ± 1.5 days (ranging from 2 to 9 days) (Table 1). Only one patient stayed 9 days post-operatively and was admitted to the intensive care unit (ICU) and re-operated, as she developed severe abdominal pain and abdominal distension and was hemodynamically unstable on the first postoperative day. During a review of her recorded LA video, a small amount of greenish fluid (bile) in the operated area was noticed (Figure 1), which was unrecognized by the surgical team. Thus, she was diagnosed with bowel injury. The patient was explored on the second postoperative day, and suturing was done to remove 0.5 cm of tear in the posteromedial surface of the second part of the duodenum.

**Table 1:** Sociodemographic and tumour characteristics of the patients

Case	Age (years)	Gender	Laterality	BMI	Diameter (mm)	Presentation	Biochemical result	Histopathology	Follow-up (months)
1	29	F	L	29	30	Incidentaloma	N	BCA	36
2	23	F	R	23	40	Incidentaloma	N	ML	33
3	56	F	R	33	42	Incidentaloma	N	ML	31
4	27	F	L	24	78	Incidentaloma	N	Benign oncocytoma	29
5	54	F	R&L	42	L70/R 68	Cushing disease	Hypercortisolism	Adrenal hyperplasia	27
6	56	M	L	40	48	Incidentaloma	N	BCA	27
7	54	F	L	39	45	Incidentaloma	N	BCA	24
8	27	F	R	32	75	Hypertension	Pheochromocytoma	BMPh	22
9	36	M	R	27	27	Conn’s syndrome	Hyperaldosteronism	BCA	20
10	44	F	L	32	50	Hypertension	Pheochromocytoma	BMPh	19
11	53	F	R	28	35	Hypertension	Pheochromocytoma	BMPh	17
12	28	F	L	23	56	Incidentaloma	N	GN	15
13	48	M	R	36	54	Hypertension	Pheochromocytoma	BMPh	12
14	36	F	L	33	44	Incidentaloma	N	BCA	12
15	28	F	L	22	54	Cohn’s syndrome	Hyperaldosteronism	BCA	10
16	39	M	R	23	42	Incidentaloma	N	BCA	7
17	34	M	L	21	53	Incidentaloma	N	ML	6
18	44	F	L	33	65	Cushing syndrome	Hypercortisolism	BCA	5
19	38	M	L	28	48	Hypertension	Pheochromocytoma	BMPh	3
20	43	M	R	23	58	Incidentaloma	N	BCA	3

M: male, F: female, N: negative, BMI: body mass index, BCA: benign cortical adenoma, ML: myelolipoma, BMPh: benign medullary pheochromocytoma, GN: ganglioneuroma.

Moreover, the maximum mass size was 7.8 cm (a case of benign oncocytoma) for nonfunctioning masses and 7.5 cm for functioning masses (a case of pheochromocytoma). Most adrenal masses were non-functioning (*n* = 11, 55%, incidentaloma; incidentally detected through radiology), while the rest were functioning (45%). Among functioning cases, five patients had pheochromocytoma (25% presented with hypertension), followed by hypercortisolism (*n* = 2, 10%, Cushing disease) and hyperaldosteronism (*n* = 2, 10%, Conn’s syndrome). Furthermore, histopathological examination revealed that most patients (*n* = 9, 45%) had benign cortical adenoma (BCA), followed by benign medullary pheochromocytoma (*n* = 5, 25%), then myelolipoma (*n* = 3, 15%). In contrast, only one case from each benign oncocytoma, adrenal hyperplasia, and ganglioneuroma was reported (5.0% each) (Table 1). Based on the Clavien–Dindo classification to rank the severity of a surgical complication, two patients had Dindo 1–2 (10%) and only one patient had Dindo 3–4 (5.0%). In contrast, no one had Dindo 5 and without any mortality among operated patients (Table 2).

**Table 2:** Clinical outcomes in our case series

Intraoperative data	Number of cases
ICU admission	1*
**Complication**	
Dindo 1–2	2**
Dindo 3–4	1*
Dindo 5	0
Re-operation	1*
Mortality	0

*Duodenal injury requiring ICU admission and reoperation. **Transient ileus.

## 4 Discussion

Choosing from the available surgical strategies to manage adrenal masses is a big challenge. Thus, we planned to share our initial experience with the LA performed by the same urologic team for the first time at Sulaimani Teaching Hospital, Sulaimaniyah, Kurdistan Region, Iraq, for patients with an adrenal mass. Finally, our results were compared to the literature (Table 3). In this study, most patients were overweight, young adults, and females, with a mean mass size of 50.65 mm, and were detected incidentally (not due to excess hormonal secretion). These results somewhat agreed with those of Iñiguez-Ariza et al., who found that older age, male sex, non-incidental mode of discovery, larger mass size, and higher unenhanced CT attenuation were associated with an increased risk for malignancy [10].

**Table 3:** Comparative clinical outcome between our case series and some other studies

Study	No. of cases	Mean operative time (min)	Mean hospitalization (days)	Conversion	Clavien-Dindo 3 and 4	Mortality
Köstek et al. [6]	76	N/A	7.1	4 (5.3%)	9 (11.8%)	1 (1.3%)
Öz et al. [7]	100	101	3.4	8 (8%)	9 (9%)	0
Simforoosh et al. [13]	65	149	N/A	5 (7.5%)	4 (5.8%)	0
Naya et al. [18]	28	202	9	4	0	0
Our case series	20	95	3.2	0	1 (5%)	0

Moreover, in the present study, most patients had a left-side mass (55%), followed by a right-side mass (40%), and only one case (5.0%) had a bilateral mass that experienced bilateral adrenalectomy. These results align with Iñiguez-Ariza et al., who found that 43% of patients had left-side mass, 42% had right-side mass, and 15% had bilateral mass [10]. Also, we found the mean operation time of 95 ± 2.0 min (ranging from 45 to 160 min) and the mean hospital stay of 3.2 ± 1.5 days (ranging from 2 to 9 days). This team’s operation time and hospital stay were less than those reported by Di Buono et al., who found the mean operative time of 145 min (ranging from 75 to 240 min) and mean length of hospital stay of 3.7 days (ranging from 3 to 6 days) [11]. Additionally, the maximum mass size in the current study was 7.8 cm (benign oncocytoma, fourth case) for a nonfunctioning mass and 7.5 cm for a functioning mass (pheochromocytoma, eighth case). However, other studies consider a maximum mass diameter of 12 cm for LA [12,13], while others suggest LA for mass sizes up to 15 cm [14,15].

Some studies suggest open surgery for adrenal masses that are highly suspected of malignancy, recurrence, having lymphadenopathy, or vascular thrombosis [14]. Sommerey et al. published 10 years of experience in LA and suggested a traditional approach to complete resection and lymphadenectomy to achieve a long-term cure [16]. Nevertheless, Brix et al. performed both open and LA for 152 patients and found a comparable frequency of mass laceration and peritoneal contamination in both groups, with better quality of life in patients who underwent LA [17,18].

We reported no incidence of intraoperative prolonged hemodynamic instability, no intraoperative blood transfusion, no conversion to open, and no mortality. One case (5.0%) had a severe complication and reoperation. She was admitted to the ICU and re-operated on by laparotomy for bowel injury on the second postoperative day. These results are much better and superior to some other studies, such as that of Simforoosh et al., who operated 65 cases through the transperitoneal approach, and they had a 7.5% conversion rate (5 cases) and a 5.8% complication rate (4 cases) [13]. Köstek et al. reported 9 cases (11.8%) with complications in their series of 76 adrenalectomies: bleeding from the spleen (2 cases), bleeding from liver parenchyma (2 cases), one case of bleeding from the upper pole of the kidney, and one renal artery injury [6]. Öz et al. reported a 9.0% complication rate among 100 cases, of which eight patients suffered intraoperative bleeding that converted to open surgery, and one case had a diaphragmatic rupture [7].

## 5 Conclusions

LA is a safe, adorable, and effective option for selected cases in our locality. Our surgical team’s outcome is comparable to other series in the literature in terms of a short operative time, low blood loss, no conversion to open surgery, and a short hospital stay.

## References

[1] Crona J, Beuschlein F, Pacak K, Skogseid B. Advances in adrenal tumors 2018. Endocr-Relat Cancer. 2018;25:R405–20. 10.1530/ERC-18-0138 PMC5976083 29794126

[2] Bancos I, Prete A. Approach to the patient with adrenal incidentaloma. J Clin Endocrinol Metab. 2021;106:3331–53. 10.1210/clinem/dgab512 PMC8530736 34260734

[3] Jing Y, Hu J, Luo R, Mao Y, Luo Z, Zhang M, et al. Prevalence and characteristics of adrenal tumors in an unselected screening population: a cross-sectional study. Ann Intern Med. 2022;175:1383–91. 10.7326/M22-1619 36095315

[4] Fassnacht M, Tsagarakis S, Terzolo M, Tabarin A, Sahdev A, Newell-Price J, et al. European Society of Endocrinology clinical practice guidelines on the management of adrenal incidentalomas, in collaboration with the European Network for the Study of Adrenal Tumors. Eur J Endocrinol. 2023;189:G1–42. 10.1093/ejendo/lvad066 37318239

[5] Bhargava P, Sangster G, Haque K, Garrett J, Donato M, D’Agostino H. A multimodality review of adrenal tumors. Curr Probl Diagn Radiol. 2019;48:605–15. 10.1067/j.cpradiol.2018.10.002 30472137

[6] Köstek M, Aygün N, Uludağ M. Laparoscopic approach to the adrenal masses: single-center experience of five years. Şişli Etfal Hastanesi Tip Bülteni. 2020;54:52–7. 10.14744/SEMB.2019.40225 PMC7192254 32377134

[7] Öz B, Akcan A, Emek E, Akyüz M, Sözüer E, Akyıldız H, et al. Laparoscopic surgery in functional and nonfunctional adrenal tumors: A single-center experience. Asian J Surg. 2016;39:137–43. 10.1016/j.asjsur.2015.04.009 26170103

[8] Arolfo S, Giraudo G, Franco C, Parasiliti Caprino M, Seno E, Morino M. Minimally invasive adrenalectomy for large pheochromocytoma: not recommendable yet? Results from a single institution case series. Langenbeck’s Arch Surg. 2022;407:277–83. 10.1007/s00423-021-02312-8 PMC8847286 34468864

[9] Almeida CEC, Caroço T, Silva MA, Albano MN, Louro JM, Carvalho LF, et al. Posterior retroperitoneoscopic adrenalectomy – Case series. Int J Surg Case Rep. 2018;51:174–7. 10.1016/j.ijscr.2018.08.044 PMC6122227 30173077

[10] Iñiguez-Ariza NM, Kohlenberg JD, Delivanis DA, Hartman RP, Dean DS, Thomas MA, et al. Clinical, biochemical, and radiological characteristics of a single-center retrospective cohort of 705 large adrenal tumors. Mayo Clin Proc: Innovations, Qual Outcomes. 2018;2:30–9. 10.1016/j.mayocpiqo.2017.11.002 PMC6124341 30225430

[11] Di Buono G, Buscemi S, Lo Monte AI, Geraci G, Sorce V, Citarrella R, et al. Laparoscopic adrenalectomy: preoperative data, surgical technique and clinical outcomes. BMC Surg. 2019;18:1–7. 10.1186/s12893-018-0456-6 PMC7402565 31074390

[12] Gagner M, Pomp A, Heniford BT, Pharand D, Lacroix A. Laparoscopic adrenalectomy: lessons learned from 100 consecutive procedures. Ann Surg. 1997;226:238–47. 10.1097/00000658-199709000-00003 PMC1191015 9339930

[13] Simforoosh N, Soufi MH, Basiri A, Ziaei S, Behjati S, Mohammad ABF, et al. Laparoscopic adrenalectomy ten-year experience, 67 procedures. Tehran, Iran: Urology and Nephrology Research Center; 2008. 18454428

[14] Ali JM, Liau S-S, Gunning K, Jah A, Huguet EL, Praseedom RK, et al. Laparoscopic adrenalectomy: auditing the 10 year experience of a single centre. Surg. 2012;10:267–72. 10.1016/j.surge.2011.08.003 22959160

[15] Agrusa A, Romano G, Frazzetta G, Chianetta D, Sorce V, Di Buono G, et al. Laparoscopic adrenalectomy for large adrenal masses: single team experience. Int J Surg. 2014;12:S72–4. 10.1016/j.ijsu.2014.05.050 24862666

[16] Sommerey S, Foroghi Y, Chiapponi C, Baumbach SF, Hallfeldt KK, Ladurner R, et al. Laparoscopic adrenalectomy – 10-year experience at a teaching hospital. Langenbeck’s Arch Surg. 2015;400:341–7. 10.1007/s00423-015-1287-x 25721680

[17] Brix D, Allolio B, Fenske W, Agha A, Dralle H, Jurowich C, et al. Laparoscopic versus open adrenalectomy for adrenocortical carcinoma: surgical and oncologic outcome in 152 patients. Eur Urol. 2010;58:609–15. 10.1016/j.eururo.2010.06.024 20580485

[18] Naya Y, Nagata M, Ichikawa T, Amakasu M, Omura M, Nishikawa T, et al. Laparoscopic adrenalectomy: comparison of transperitoneal and retroperitoneal approaches. BJU Int. 2002;90:199–204. 10.1046/j.1464-410x.2002.02845.x 12133053

